# Noradrenaline for progressive supranuclear palsy syndromes (NORAPS): a randomised, double-blind, placebo-controlled, crossover Phase IIb clinical trial evaluating the efficacy and safety of oral atomoxetine for treating cognitive and behavioural changes in people with progressive supranuclear palsy syndromes in the UK

**DOI:** 10.1136/bmjopen-2025-099577

**Published:** 2025-07-28

**Authors:** Robert Durcan, Hugo Paula, Boyd C P Ghosh, Duncan Street, Juliet High, Colin McAlister, Lee Shepstone, Charlotte Russell, Kelly Grant, Natalia Igosheva, Christopher T Rodgers, Simon P Jones, Rong Ye, Christopher Kobylecki, Alistair Church, Chrystalina Antoniades, Vicky Marshall, Luca Passamonti, James B Rowe

**Affiliations:** 1 Department of Clinical Neurosciences, University of Cambridge, Cambridge, UK; 2 Cambridge University Hospitals NHS Foundation Trust, Cambridge, UK; 3 Wessex Neurological Centre, University Hospital Southampton NHS Foundation Trust, Southampton, UK; 4 Norwich Clinical Trials Unit, University of East Anglia, Norwich, UK; 5 Cambridge Clinical Trials Unit, Cambridge University Hospitals NHS Foundation Trust, Cambridge, UK; 6 Manchester Centre for Clinical Neurosciences, Northern Care Alliance NHS Foundation Trust, Salford, UK; 7 Royal Gwent Hospital, Newport, UK; 8 Nuffield Department of Clinical Neurosciences, University of Oxford, Oxford, UK; 9 Institute of Neurological Sciences, Queen Elizabeth University Hospital, Glasgow, UK; 10 MRC Cognition and Brain Sciences Unit, Cambridge, UK

**Keywords:** Noradrenaline, PSP, Apathy, Impulsivity, Atomoxetine

## Abstract

**ABSTRACT:**

**Introduction:**

Progressive supranuclear palsy (PSP) is a devastating neurodegenerative disease characterised by cognitive, behavioural and motor problems. Motor symptoms are highly disabling, while cognitive and behavioural changes have a major impact on carer burden, quality of life and prognosis. Apathy and impulsivity are very common, often coexistent in PSP, and negatively predict survival. In preclinical models and other diseases, apathy and impulsivity are associated with noradrenergic deficits, which can be severe in PSP.

**Methods and analysis:**

Noradrenaline for Progressive Supranuclear Palsy Syndromes trial is a randomised, double-blind, placebo-controlled, crossover design, Phase IIb clinical trial to evaluate the efficacy and safety of oral atomoxetine for the treatment of cognitive and behavioural changes in PSP. Participants receive atomoxetine 40 mg (10 mg/mL oral solution) once daily or a matched placebo solution, in random order, each for 8 weeks. An ‘informant’, who knows the patient with PSP well, is co-recruited to complete some of the trial outcome measures. Participants remain in the trial for 22 weeks after randomisation. The primary objectives are to assess (1) safety and tolerability and (2) efficacy versus placebo on challenging behaviours as reported in a subscale of the Cambridge Behavioural Inventory. Secondary and exploratory measures relate to cognition, the PSP Rating Scale, mood and potential baseline predictors of individual response to atomoxetine computed from imaging, genetic and cognitive measures at baseline.

**Ethics and dissemination:**

The trial was approved by the South Central-Oxford B Research Ethics Committee (REC) and the Medicines and Healthcare products Regulatory Agency (REC reference: 20/SC/0416). Dissemination will include publication in peer-reviewed journals, presentations at academic and public conferences and engagement with patients, the public, policymakers and practitioners.

**Trial registration number:**

ISRCTN99462035; DOI: https://doi.org/10.1186/ISRCTN99462035; EudraCT (European Union Drug Regulating Authorities Clinical Trials Database)/CTIS (Clinical Trial Information System) number: 2019-004472-19; IRAS (Integrated Research Application System) number: 272063; Secondary identifying numbers: CPMS (Central Portfolio Management System) 44441.

**STRENGTHS AND LIMITATIONS OF THIS STUDY**

- Multisite recruitment with broad inclusion criteria, including multiple phenotypical variants of progressive supranuclear palsy (PSP).
- Outcomes draw on clinician-reported, carer-reported and patient-reported evaluations.
- Collection and analysis of blood and imaging biomarkers offer potential windows to understand the biological underpinnings of cognition and behaviour in PSP.
- MRI scanning at baseline is requested but not essential to participate.
- The trial recruits from an elderly population in the UK, where the majority of candidate participants are white, potentially limiting the global generalisation of findings.

## Introduction

 Progressive supranuclear palsy (PSP) is a devastating neurodegenerative disease characterised by a series of cognitive, behavioural and motor problems.^1 2^ The prevalence of people with a diagnosis of PSP is approximately 5–7 per 100 000 people.^3 4^ Motor symptoms are highly disabling, while cognitive and personality changes also affect patients’ and carers’ quality of life (QoL).^5 6^ Apathy and impulsivity are common and often coexist in PSP, and are positively correlated.^7^ They cause substantial patient morbidity and carer distress^28^,^10^ and are associated with poor prognosis.^11 12^ Despite their significant impact on the QoL of patients with PSP and carers alike, the presence and impact of apathy and impulsivity remain poorly recognised and treated in clinical settings.

The underlying causes of apathy and impulsivity in PSP are multifactorial.^13^ Dopaminergic medications, which are sometimes useful for the treatment of motor symptoms, are largely ineffective for the treatment of apathy and impulsivity in PSP and may even worsen impulsive behaviours.^14^ However, there is converging evidence that alterations in the noradrenergic system play a fundamental role in mediating both apathy and impulsivity.^7 15^

PSP is associated with early and severe loss of neurons in the locus coeruleus (LC), the main source of noradrenaline (NA) in the brain.^15^ NA directly affects the cognitive and neural processes involved in stopping actions, delay tolerance^16 17^ and the regulation of motivated behaviours.^18^ This raises the hypothesis that challenging behaviours with apathy and impulsivity in PSP are in part due to depletion of NA in the forebrain following neurodegeneration of the LC. Indeed, blocking NA reuptake improves response inhibition in both animals^19^ and humans,^20^,^22^ as well as measures related to apathy.^23^

Atomoxetine is a selective NA reuptake inhibitor (SNRI) currently licensed for the treatment of children with attention-deficit/hyperactivity disorder (ADHD). In line with studies of ADHD,^20^ our group and others have highlighted the potential for atomoxetine to treat impulsivity in Parkinson’s disease (PD), enhancing connectivity in prefrontal networks and improving response inhibition.^2224^,^26^ The noradrenergic deficit in PSP is more severe than in PD, with up to 90% loss of the NA-producing cells in the brain (mean 49% loss).^27^ Noradrenergic drugs have also emerged as a potential treatment for apathy in Alzheimer’s disease.^28^

In this clinical trial, we aim to treat apathy and impulsivity jointly. We test the hypothesis that the severe noradrenergic deficit in PSP, arising from degeneration of the LC, contributes to both apathy and impulsivity. The critical predictions are that atomoxetine treatment is well tolerated for people with PSP and mitigates apathy and impulsive behaviours as observed by caregivers.

### Aims and objectives

The NA for PSP Syndromes trial (NORAPS) aims to assess the efficacy and safety of atomoxetine for the treatment of challenging behaviours (including apathy and impulsivity) in people with PSP, using a standard dose (40 mg oral solution daily for 8 weeks), in a multicentre, crossover-design, placebo-controlled, randomised clinical trial.

#### Primary objective

To (1) demonstrate the *safety* and *tolerability* in people with PSP, and (2) estimate (with inferences) the *efficacy* of atomoxetine versus placebo for the treatment of apathy and impulsivity, as measured by the Cambridge Behavioural Inventory Revised (CBI-R) apathy and impulsivity composite score.

#### Secondary objectives

To assess the group-level effect of atomoxetine compared with placebo on:

- Global clinical improvement measured using the Clinical Global Impression of Change (CGI-C) at 8 and 18 weeks and the CGI of Severity (CGI-S) at 0 and 10 weeks.^29^
- Behaviour and cognition measured using the CBI-R total score and the Cambridge Questionnaire for Apathy and Impulsivity Traits (CamQUAIT) apathy score, completed by the research partner at 0, 8, 10, 18 and 22 weeks.^30^
- Cognitive and behavioural functioning measured using the Conners’ Adult ADHD Rating Scale (CAARS) Short Form subscores, completed by both the patient and research partner at 0, 8, 10, 18 and 22 weeks.^31^
- Anxiety and depression measured using the patient-rated Hospital Anxiety and Depression Scale (HADS) at 0, 8, 10, 18 and 22 weeks.^32^
- Patient and carer (research partner) QoL measured using the PSP QoL scale (PSP-QoL)^5^ and the Parkinsonism QoL for carers (PQoL Carers),^6^ respectively, at 0, 8, 10, 18 and 22 weeks.
- General measures of cognitive functioning measured using the clinician-rated Repeatable Battery for the Assessment of Neuropsychological Status (RBANS), which includes verbal fluency, at 0, 8, 10 and 18 weeks.^33^
- Motivation-directed and goal-directed behaviour, using the motivation subscore of the CBI-R.^34^

#### Exploratory objectives

NORAPS will explore the effect of atomoxetine compared with placebo on:

- Severity of PSP and disease progression as measured by the PSP Rating Scale (PSPRS)^35^ at 0, 8, 10 and 18 weeks.
- Baseline clinical deficit measured using the clinician-rated PSP Clinical Deficit Scale (PSP-CDS) at baseline.^36^
- Cognition, as measured by the Montreal Cognitive Assessment (MoCA)^37^ at 0, 8, 10 and 18 weeks.
- Executive function, as measured by the Frontal Assessment Battery (FAB)^38^ at 0, 8, 10 and 18 weeks.
- Response inhibition, as measured by performance on the stop signal task (SST)^39^ at 0, 8, 10 and 18 weeks.
- Heart rate variability, as measured by an ECG undertaken 1–3 hours after the initial dose of atomoxetine at 0 and 10 weeks.
- Treatment response and tolerability measured using plasma levels of atomoxetine at 0 and 8 or 10 and 18 weeks (depending on arm of crossover study).

NORAPS will evaluate the effect of atomoxetine as a function of individual differences in:

- Genetic variations, including but not limited to cytochrome P450 2D6 (CYP2D6) polymorphism (poor metaboliser vs extensive metaboliser)^40^ and noradrenaline transporter gene (NET/SLC6A2) polymorphism.^41^
- Plasma levels of atomoxetine (visits 2–5; measured approximately 2 hours after ingestion on visits 2 and 4).
- Structural integrity of the brain, including LC (the main source of brain NA), as measured by high-field (3T) and ultra-high field (7T) MRI imaging.

## Methods and analysis

### Trial design

**Figure 1:**
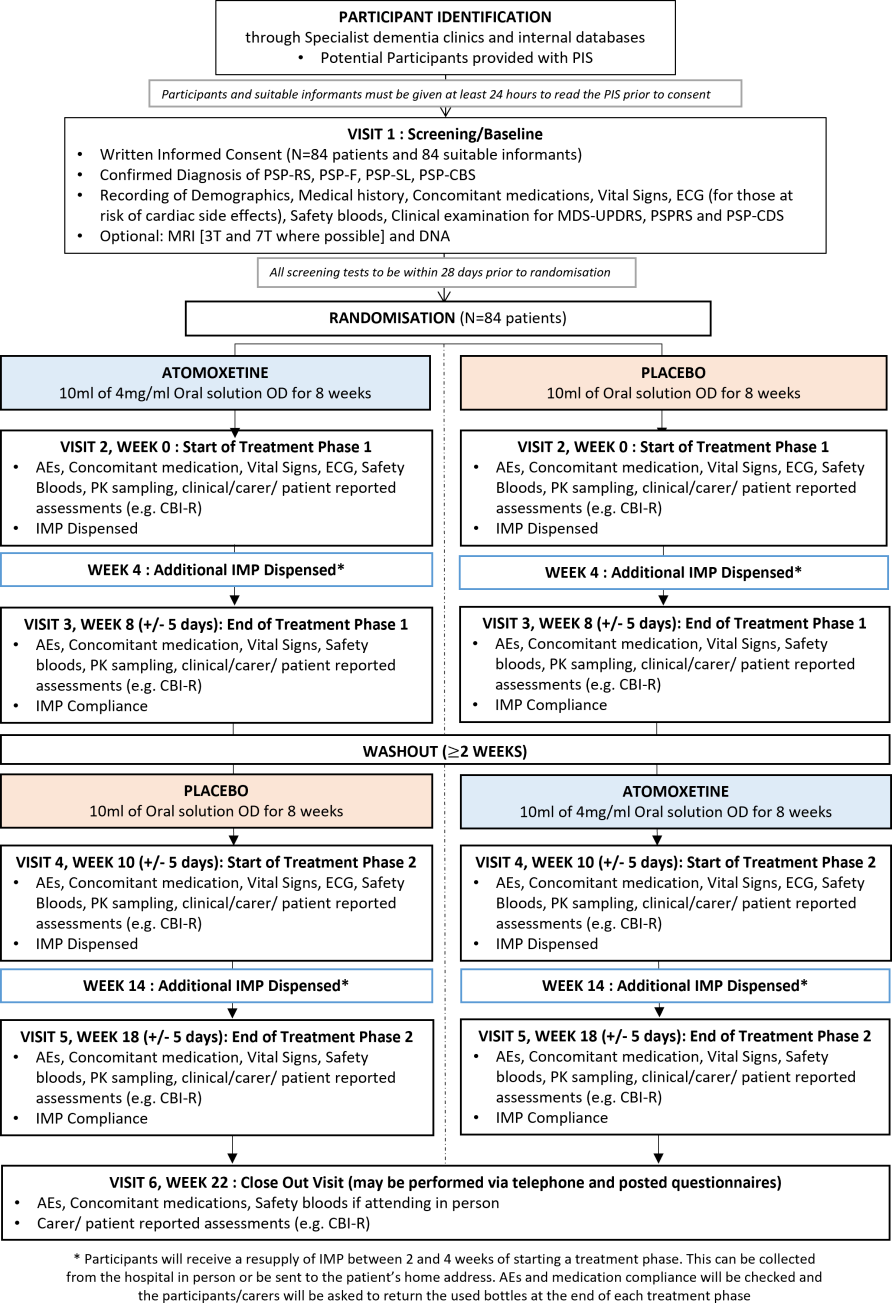
Illustration of trial design and participants’ journey. Participation in the trial is based on six trial site visits. *Participants will receive a resupply of IMP between 2 and 4 weeks of starting a treatment phase. This can be collected from the hospital in person or be sent to the patient's home address. AEs and medication compliance will be checked and the participants/carers will be asked to return the used bottles at the end of each treatment phase. PIS, Patient Information Sheet; N, number of participants; AEs, adverse events; IMP, investigational medicinal product; OD, once daily; MDS-UPDRS, Movement Disorder Society-Unified Parkinson’s Disease Rating Scale; PSP-CBS, progressive supranuclear palsy with predominant corticobasal syndrome; PSP-CDS, Progressive Supranuclear Palsy Clinical Deficit Scale; PSP-F, progressive supranuclear palsy with predominant frontal presentation; PSP-RS, progressive supranuclear palsy-Richardson syndrome; PSP-SL, progressive supranuclear palsy with predominant speech/language disorder; PSPRS, progressive supranuclear palsy rating scale.

NORAPS is a Phase IIb randomised, placebo-controlled crossover study using atomoxetine, a SNRI, in people with PSP. A standard dose of 40 mg once daily is used in liquid formulation. A bespoke-manufactured placebo is used, matched for taste, colour and consistency, formulated by Guy’s and St Thomas’ NHS Foundation Trust Pharmacy Manufacturing Unit, London, UK. The crossover design ensures that all participants receive the drug at some point through the trial and act as their own control. The trial design is outlined in figure 1.

Trial sites undertake a prescreening of their known patient population to identify and invite potentially suitable participants, along with an informant, who are provided with written information sheets, and invited to a screening visit. Other permitted modes of recruitment include direct referral to the study team at a trial site, with national awareness of the trial facilitated through the PSP Association, Parkinson’s UK and local patient-based support networks.

### Population

Key inclusion criteria

1. Participants must have the mental capacity to give informed consent.
2. Diagnosis of possible or probable PSP, including the PSP-Richardson Syndrome (PSP-RS), PSP with predominant frontal presentation, PSP with predominant corticobasal syndrome or PSP with predominant speech/language disorder variants under the 2017 Movement Disorder Society (MDS) diagnostic criteria for PSP.^10^
3. Male or female patient, aged 50–85 years.
4. Have a ‘research partner’, who has a minimum of weekly telephone or face-to-face contact, is able and consents to provide information on proxy measures (eg, a relative, unpaid or paid carer or care home manager).
5. Stable psychoactive medications for at least 28 days from visit 1, with no known plans to change medication. No ramping up or weaning off medications, including levodopa, dopaminergic agonists, anticholinergics, amantadine, other antiparkinsonian medications or antidepressants or any other psychoactive medication.
6. Be able to participate in the study.
7. Female patients must be surgically sterile or be postmenopausal. A postmenopausal state is defined as no menses for 12 months without an alternative medical cause. A high follicle-stimulating hormone (FSH) level in the postmenopausal range may be used to confirm a postmenopausal state in women not using hormonal contraception or hormonal replacement therapy. However, in the absence of 12 months of amenorrhoea, a single FSH measurement is insufficient. Surgically sterile is defined as women who have had a hysterectomy, bilateral salpingectomy or bilateral oophorectomy at least 6 weeks prior to enrolment. Premenopausal female patients can still be included but will undergo pregnancy tests (at screening and at all four treatment visits) to confirm that they are not pregnant.

Research partner inclusion criteria

1. A minimum of once-a-month face-to-face or telephone contact with the main study participant.
2. Able to provide information on proxy measures.
3. Aged ≥16 years.

Exclusion criteria

1. Use of a monoamine oxidase inhibitor, SNRI or other drugs that alter monoamine concentrations (including tricyclic antidepressants with the exception of low-dose amitriptyline ≤30 mg) within 2 weeks of visit 1, or a known clinical care plan to introduce such treatment.
2. Use of high dose (>10 mg) of systemic steroids (eg, prednisolone).
3. Significant cardiovascular disease, such as ischaemic heart disease or cardiac rhythm abnormalities.
4. Narrow (acute) angle glaucoma.
5. A history of, or current, phaeochromocytoma.
6. Known significant hepatic or renal impairment (Alanine transaminase (ALT) or aspartate transaminase (AST) over three times the reference range and total bilirubin>2 times the reference range; estimated glomerular filtration rate (eGFR)<45 mL/min and/or serum creatinine>168µmol/L (1.9 mg/dL)). This threshold of renal impairment is higher than the common clinical definition of renal ‘failure’ of eGFR of 15 mL/min. Note that at the initial registration of the trial, the eGFR threshold was <60 mL/min, but this has been reduced by an approved amendment to the protocol.
7. A medical condition that the principal investigator (PI) feels may interfere with the participant’s ability to comply with study instructions would place the participant at increased risk or might confound the interpretation of the study results.
8. History of cancer within 3 years of visit 1, with the exception of fully excised non-melanoma skin cancers or non-metastatic prostate cancer.
9. Presence of significant neurological (other than PSP) or psychiatric disorders, including any psychotic disorder, clinically significant depression or suicidal thoughts or behaviour that are believed by the PI to represent a current safety risk (including a seizure within 3 years of visit 1 or a history of recurrent seizures).
10. Known presence of known disease-associated mutation in genes known as C9ORF72, GRN, CHMP2B, TBK1, TARBP or VCP or any other frontotemporal lobar degeneration causative genes not associated with underlying τ pathology (individuals with MAPT mutations may participate if they meet all other eligibility criteria).
11. Any major surgery within 28 days of visit 1.
12. Evidence of organ dysfunction or any clinically significant deviation from normal function in physical examination (neurological examination, vital signs and ECG) or clinical laboratory determinations beyond what is consistent with the target population.
13. Recent (in the last month) or current systemic infections.
14. Current participation in any other clinical trial of an investigational medicinal product (IMP).
15. Likely inability to give blood, for example, needle phobia.
16. Insufficient proficiency in English to provide informed consent as assessed by the clinical or study team.
17. Body weight outside the range of 40–120 kg.
18. Breastfeeding female.
19. Any other contraindication to atomoxetine treatment as detailed in the atomoxetine *summary of product characteristics* documentation, including but not limited to hypersensitivity to the active substance or to any of the excipients.

Note: contraindication(s) to MRI is not an exclusion criterion for trial participation.

#### Screening visit

The first visit begins with written informed consent. Participants and research partners provide consent to a member of the research team using the dedicated information sheet and consent form (see online supplemental material). This is followed by the assessment of medical history and examination to confirm the diagnosis and inclusion and exclusion criteria. This visit includes the MDS-Unified PD Rating Scale (MDS-UPDRS) and the PSPRS, from which full-scale and modified subscale values are derived, as well as the PSP-CDS. Medical history, demographics, body weight, pulse rate and blood pressure, as well as concomitant medications, are recorded. Safety assessments also include blood tests via venepuncture for renal and hepatic function, FSH and/or pregnancy test if required, the Columbia Suicide Severity Rating Scale (C-SSRS) and a 12-lead ECG. Blood is also taken for COVID-19 status and genetic analysis at trial conclusion to assess for CYP2D6 and NA transporter gene polymorphisms, NET/SLC6A2.

High-resolution MRI scans are acquired using 7T MRI scanners where possible, or 3T MRI scanners where this is not available. Both genetics and brain imaging are encouraged but are optional for participants.

A whole-brain 0.7 mm isotropic Magnetization-Prepared 2 Rapid Acquisition Gradient Echo (MP2RAGE) sequence was acquired using the UK7T Network harmonised protocol (Clarke *et al*)^42^:Repetition time (TR)=3500 ms, Echo Time (TE) =2.59 ms, bandwidth (BW)=300 Hz/px, voxel size=0.7×0.7×0.7 mm, Fielf of View (FoV)=224×224×157 mm, acceleration factor (A>>P)=3, flip angles=5/2° and inversion times (TI)=725/2150 ms for the first/second images. A magnetisation transfer (MT)-sensitised TurboFLASH (TFL) sequence to quantify the loss of neuromelanin-containing cells covering LC to substantia nigra was acquired using a 3D high-resolution MT-TFL sequence for imaging the LC (Priovoulos *et al*)^43^ comprising 112 axial slices with a train of 20 Gaussian-shaped radiofrequency (RF) pulses at 6.72 ppm off-resonance, 420° flip angle, followed by a TurboFLASH readout (TE=4.08 ms, TR=966 ms, flip angle=8°, voxel size=0.4×0.4×0.5 mm^3^, 6/8 phase and slice partial Fourier, BW=140 Hz/px, no acceleration and 14.3% oversampling). An additional MT scan was acquired with the same parameters as above but with a TR of 1250 ms and without the off-resonance pulses to directly allow the examination of the MT effect. For each subject, the transmit voltage was adjusted based on the average flip angle in the central area of the pons, obtained from a B1 precalibration scan. To assess iron accumulation, a multiecho T2*-weighted sequence was used with a 1.4 mm isotropic resolution, TE1=4.68 ms, 6 echoes with echo spacing 3.24 ms, TR=27 ms and nominal fractional anisotropy (FA) =15°. A functional resting-state scan sequence was acquired using a 2D Gradient-Echo Echo-Planar Imaging sequence, with TR=1589 ms, TE=24.8 ms, FoV=192×192 mm^2^, flip angle=70°, resolution=1.13×1.13×1.10 mm^3^, number of slices=110, partial Fourier=6/8, Generalized Autocalibrating Partially Parallel Acquisitions (GRAPPA) factor=2, multiband factor=5, BW=1838 Hz/px and echo spacing=0.78 ms.

The sequences acquired at 3T include a whole-brain sagittal MP2RAGE (TR=2000 ms, TE=2.85 ms, BW=240 Hz/px, voxel size=1.1×1.1×1.1 mm, FoV=282×282 mm, 229 slices, acceleration factor (A>>P)=2 and flip angle=8°) and a T2 space volume (TR=3200 ms, TE=401 ms, BW=751 Hz/px, voxel size=1.1×1.1×1.1 mm, FoV=282×282 mm, 176 slices, acceleration factor (A>>P)=2 and flip angle=120°). For resting-state functional MRI, echo planar imaging was acquired with 200 volumes, voxel size=3.0×3.0×3.5 mm, FoV=192×192 mm, 42 slices, slice thickness=3.5 mm, TR=2500 ms, TE=30 ms and flip angle=80°. A single-shell diffusion-weighted sequence was acquired with 64 gradient directions (b=1000 s/mm^2^) and 5 B0 (b=0 s/mm^2^) volumes, with isotropic voxel size=2.5 mm, FoV=240×240 mm, 59 slices, slice thickness=2.5 mm, TR=7300 ms, TE=90 ms and GRAPPA factor=2. Additionally, a multishell diffusion-weighted sequence was acquired for the neurite orientation dispersion and density imaging (NODDI) pipeline comprising 117 volumes with 13 non-diffusion-weighted images (b=0 s/mm^2^) and 104 diffusion-encoding gradient direction volumes (b=300, 700 and 2000 s/mm^2^), with isotropic voxel size=2.0 mm, FoV=220×220 mm, 59 slices, slice thickness=2.0 mm, TR=3350 ms, TE=58 ms and GRAPPA factor=2. A pulsed arterial spin labelling 3D gradient-and-spin-echo sequence was acquired with five averages comprising a labelling duration of 800 ms, a postlabelling delay of 1200 ms, TR=4000 ms, TE=13.22 ms, FoV=240×240 mm^2^, slice thickness=4.5 mm, flip angle=130°, voxel resolution=3.75×3.75×4.00 mm^3^ and sampled to 3.75×3.75×4.50 mm^3^.

The screening visit leads to confirmation of eligibility status. Eligible people proceed to randomisation of treatment order within 28 days. Visit 2 should occur at the earliest convenience, but no later than 14 days postrandomisation.

#### Visits 2–5

Visits 2–5 follow a structured format, including the following assessments:

1. Clinical status and safety assessment, updating on concomitant medication and any adverse events (AEs) not already reported. AEs are recorded using the Common Terminology Criteria for AE framework (V.5).
2. C-SSRS.
3. First dosing of active compound or placebo (start of visits 2 and 4 only).
4. Endpoints reported or assessed directly with the patient with PSP include PSP-QoL, CAARS, HADS, PSPRS, Stop/NoGo task, FAB, RBANS and MoCA.
5. Endpoints based on informant reports include CBI-R, CamQUAIT, CAARS informant and PQoL Carers.
6. CGI scales include CGI-S at visits 2 and 4 and CGI-C at visits 3 and 5.
7. Blood for plasma biomarker and pharmacokinetic analysis, as well as renal and liver function profiles. 2×4.5 mL vials of blood are collected at each study visit. For visits 2 and 4, blood collection takes place 2 hours after dosing to minimise repeated venepuncture while facilitating pharmacokinetic analysis.
8. ECG for heart rate variability at 1 and 3 hours after dosing, respectively (start of treatment visits 2 and 4 only).

#### Visit 6 (close-out visit)

This takes place 4 weeks after the last dose of atomoxetine/placebo (approximately week 22) to monitor for delayed AEs and provide an opportunity for qualitative participant feedback on the trial participation experience.

1. Safety assessment (safety blood tests, urine pregnancy test, vital signs and, if clinically indicated, neurological examination, concomitant medication and C-SSRS) and AE recording.
2. Secondary endpoints (CBI-R, PSP-QoL informant and patient, CAARS informant and patient, CamQUAIT and HADS).
3. If the participant is unable or unwilling to attend visit 6 in person, a telephone contact visit is offered instead.

### Study setting

There are multiple participation sites around the UK (including Scotland, Wales and England). These are located in secondary or tertiary care clinics for movement disorders and/or cognitive disorders. All study visits occur at the trial site, unless visit 6 is undertaken remotely.

### Patient and public involvement

NORAPS was proposed in outline at the foundation of the Cambridge Centre for Parkinson-Plus (CCPP). The CCPP established a panel of patients and relatives with lived experience of PSP and corticobasal syndrome, to discuss and review new research proposals, their aims, scope and participant-facing documentation. The group was led by a senior research nurse who is not part of the NORAPS team. The NORAPS draft protocol and documentation were reviewed and revised in response to feedback from people with lived experience of PSP and CBD. This panel did not include participants or relatives of participants in the trial. The study progress has been presented at annual meetings of the Cambridge Neuroscience Clinical Trial Unit Annual Events 2022, 2023 and 2024, which include trial participants and individuals with lived experience of dementia and neurodegenerative disease.

#### Blinding

This is a crossover, double-blind, placebo-controlled, fixed-dose design. Participants receive 8 weeks of atomoxetine and 8 weeks of placebo, with the order of treatment randomised. Participants, their informants (and other carer/family members) and the clinical trial team (including the PI, chief investigator, trial manager and study nurses) are blinded to the treatment allocation to minimise the risk of bias. Site pharmacies will be unblinded.

Eligible consented patients will be randomised on a 1:1 basis to one of the two trial crossover arms using a web-based randomisation process with the randomisation schedule consisting of blocks of random length (2 or 4) to ensure balanced randomisation during the course of the trial.

At randomisation, an unblinded email is sent to the local site pharmacy for the patient, who, on receipt of a trial prescription, prepares the relevant treatment pack prior to the participant receiving their medication. A blinded email is sent to the trial and local research teams, confirming that randomisation has occurred.

Due to potential sedimentation in oral solutions, which may affect the appearance of the IMP (but not safety), there are additional mitigating actions to maintain blinding. The IMP bottles are dark. Participants are notified that sedimentation ‘opacity’ may occur and is not a risk to them and does not need to be reported to the study team. When unused IMP is returned to the study site at the end of each treatment phase, it is returned in opaque envelopes. If the receiving staff member is informed of sedimentation or opacification, they record this but do not report the information to other local site team members or to the lead site during the conduct of the study. If enquiries need to be made about IMP appearance, a consultant neurologist has been identified, who is not part of the main study team, to field initial enquiries. All incidences of potential or perceived unblinding will be recorded and monitored in a way that does not lead to sharing potential unblinding information with any other members of the study team.

Members of an independent data monitoring committee (DMC) will review blinded data on a regular basis to enable safety monitoring. If they have concerns, they can request unblinded information from the treatment group. This unblinded information is not disclosed to other members of the team. The DMC will comprise two external clinical experts and an independent statistician, as well as the trial statistician and statistical lead.

For the purposes of study blinding, active IMP (atomoxetine) and matched placebo will be dispensed in matched bottles and labelled with blinded Annex 13-compliant labels (approved by the Medicines and Healthcare products Regulatory Agency (MHRA)). Assembly activities (ie, rebottling in order to blind trial medication) will be undertaken by local pharmacy teams out of sight of any blinded site staff or the patient/informant/carer, friend or family member. The product will be dispensed by and returned directly to pharmacy so that research staff do not see the bottles or contents, thereof, to minimise any potential risk of unblinding.

An investigator or physician may unblind only in the case of (1) a medical emergency or in the event of a serious medical condition, and (2) when knowledge of the investigational product is essential for the clinical management or welfare of the participant. If a suspected unexpected serious adverse event is reported, delegated members of the clinical trial unit (not including the direct trial team) will be able to unblind a participant to enable expedited reporting to the regulatory agencies.

#### Intervention

Atomoxetine is an NA reuptake inhibitor, currently licensed for the treatment of ADHD in children, adolescents and adults. The 40 mg dose is at the lower end of the clinical dose range (40–100 mg). This dose of atomoxetine has also been well tolerated in psychopharmacological studies in older adults and people with sleep apnoea and PD.^21 22 24^ Doses up to 100 mg/day have been used in trials with mild cognitive impairment or for PD in older adults and were well tolerated.^44^

Atomoxetine liquid solution is commercially available in a 4 mg/mL formula. It is marketed with the brand name Strattera. It is clear and colourless. In addition to atomoxetine, each millilitre also contains 32.97 mg of sorbitol, 0.8 mg of sodium benzoate, 9.8 mg of propylene glycol and 2.64 mg of sodium. Placebo liquid is produced by Guy’s and St Thomas’ NHS Foundation Trust Pharmacy and has been analysed and deemed to adequately resemble the atomoxetine solution in appearance, colour, viscosity and taste. Both atomoxetine and placebo are packaged in identical 100 mL amber glass bottles, with a 28 mm polypropylene tamper-evident, child-resistant screw cap.

### Outcomes

The primary outcomes include:

1. Safety of atomoxetine will be determined from the number of participants with serious adverse events (SAEs) and adverse events (AEs) leading to discontinuation. Tolerability will be assessed by the review of the list of all AEs and reactions (including serious) experienced by participants during the trial.
2. Efficacy of atomoxetine against the challenging behaviours quantified by a subscale of the CBI-R.^34^ This subscale includes the following CBI-R items: abnormal behaviours (6 items), ‘euphoria’ mood items (2 items), stereotypic and motor behaviours (4 items), eating habits (4 items) and motivation (5 items).

The secondary outcome measures include:

- CGI-C.^29^
- CBI-R Motivation Subscore.^34^
- CAMQUAIT.^30^
- CAARS—patient’s and carer’s versions.^31^
- HADS.^32^
- RBANS.^33^
- PSP-QoL.^5^
- PQoL Carers.^6^

The exploratory outcome measures include:

- 7T MRI (or lower field MRI for sites without 7T MRI facility).
- Genetics (DNA for assessment of variations in the CYP2D6 and NET genes).
- MoCA.
- FAB.
- Performance on the SST, quantified primarily by Stop-Signal-Reaction-Time (integration method), with additional subject-specific values for go-reaction times, attentional and trigger failures derived from the dynamic models of change analysis method.^45 46^
- PSPRS full scale (28 items).^35^
- PSPRS modified scale (14 items).^47^
- PSP-CDS.^36^
- Levels of atomoxetine in blood.
- 12-lead ECG abnormalities, heart rate and blood pressure.

### Sample size

A total of 74 participants (37 within each sequence) were estimated a priori to provide 80% power, with statistical significance set at the two-sided 5% level, to detect an effect size of one-third. That is, a difference in mean outcome between treatments of one-third the size of the SD of the individual differences. Given the crossover design, the effect size is calculated within individuals between treatments. A dropout rate of approximately 10–15% is anticipated (based on previous studies in PSP); therefore, the target recruitment is 84 people with PSP (each with an informant). However, the number of patients recruited to the trial may be adjusted according to the actual attrition rate if necessary. Patient participants who are randomised but do not start the treatment phase will be replaced.

### Retention

The criteria for a participant to leave the study are:

- Participant’s decision.
- Confirmed pregnancy.
- A serious adverse reaction (SAR).
- Decision of the physician responsible for a study participant, on appropriate medical grounds (eg, individual AEs, illness, protocol violations, significant suicidal risk or new information gained about study procedures).

If a participant chooses to discontinue the study drug phases, they will continue to be followed up as closely as possible to the follow-up schedule defined in the protocol, provided they are willing. They are invited to continue follow-up in the trial even though they no longer take the study drug. If the reason for withdrawal relates to participant safety, a follow-up safety consultation (phone call or visit, depending on severity) is conducted within the week after the participant stops the treatment. NCTU is informed of the treatment discontinuation in writing using the appropriate trial documentation.

If a participant chooses to discontinue their study drug and is willing to be followed up, but in a limited manner, they are invited to participate in a close-out visit for safety (AEs) prior to withdrawal. The safety close-out visit may consist of a telephone call at an agreed time, approximately 2 weeks after the last dosing, during which any AEs will be collected if the participant is still willing. Full withdrawal from the trial will take effect from that time. If, in addition to withdrawal from trial treatment, the participant also expresses the view that they no longer wish to be followed up, this view will be respected, and the participant will be withdrawn entirely from the trial.

### Data analysis

All clinical trial data are collected and managed using REDCap (Research Electronic Data Capture),^48 49^ a web-based electronic case report form designed and hosted by the Norwich Clinical Trials Unit. The platform is designed with audit trails and tracking of data manipulation, with automated export procedures to a range of statistical packages to assist with analysis.

Efficacy of atomoxetine will be assessed following the last patient’s last dose and the ensuing data lock. Differences in the primary efficacy outcome measures will be assessed.

Safety of atomoxetine will be determined by calculating the numbers and percentages of deaths and unique participants with SAEs and AEs leading to discontinuation. Tolerability will be assessed by the review of the list of AEs and reactions (including serious) experienced by participants during the trial.

All data will be stored on the REDCap database, on the servers based at NCTU, apart from imaging and raw data for the Stop/NoGo test. For the Stop/NoGo test, raw data are stored locally, transferred to the lead site and then analysed by the integration method before summary data are transferred to the REDCap database. Imaging data will be transferred in electronic format to the lead site for storage, quality control and quantitative analysis.

Clinical trial data will be analysed at the Norwich Clinical Trials Unit.

Data collected at baseline, that is, visits 1 and 2 before drug administration, including demographics and MRI, will be analysed by members of the study team independently of the clinical trial outcomes. Baseline analyses will focus on advancing scientific understanding of cognitive and behavioural disturbances in PSP, with particular focus on the influence of LC integrity on apathy and impulsivity, captured using 3T and 7T MRI. All study team members collecting data and performing baseline data analysis are blinded to treatment group allocation.

Statistical analyses will be carried out to determine:

- An estimate (with inferences) of the effect of atomoxetine on cognition and behaviour, specifically whether atomoxetine is effective in reducing apathy, impulsivity and improving behavioural control.
- The influence of genetic variations in the NET gene in predicting response to atomoxetine.
- The influence of CYP2D6 on atomoxetine metabolism (nuisance variable) in predicting response to atomoxetine and in affecting pharmacokinetic measures.
- Whether volumetric differences in the LC are predictive of atomoxetine response and whether participants with significant behavioural impairments at baseline have significantly different LC volumes than participants with less severe impairments.
- To develop a predictive model of atomoxetine response by cross-validation, combining clinical and imaging features.^26^

The primary efficacy endpoint will be evaluated by comparing the cognitive and behavioural changes following 8 weeks of daily atomoxetine versus 8 weeks of placebo, as measured by the Apathy-Impulsivity composite subscore from the CBI-R. This subscore includes the following CBI-R subscales: abnormal behaviours (6 items), ‘euphoria’ mood items (2 items), stereotypic and motor behaviours (4 items), eating habits (4 items) and motivation (5 items).^50^,^52^ Owing to this being a crossover trial where participants act as their own control, the effect size is calculated within individuals between treatments (drug and placebo).

The estimate of efficacy will be through a general linear model, with the assumption of normally distributed errors. The model-dependent variable will be the difference in CBI-R between the control period and the intervention period. The model will include recruiting site (as a random effect) and the difference in CBI-R baselines. Other genuine baseline values (ie, where there can be an observable difference between the control and intervention baselines) will be considered for prognostic variables and included in the model. The intercept estimate from the model then serves as the estimate of efficacy. This will be presented with 95% CIs, and a two-sided 5% significance level will be set.

The primary analysis will follow the Intention-to-Treat principle. All participants who complete visit 2 (dose initiation) will be included, as far as data allows, irrespective of actual intervention or dosage received.

Missing values will be dealt with using the following strategy. If the degree of outcome data missing is less than 2.5%, only a complete case analysis will be used. In the case that more than 2.5% and less than 50% of data are missing initially, any missing questionnaire item data will be imputed according to any questionnaire-specific approaches; for example, if one item is missing, the mean of those completed may be used. In the absence of specific guidelines, where individual items are missing, an approach will be discussed and agreed on with the study DMC. When entire questionnaire data are missing, a multiple imputation approach will be used to impute the between-period differences. In the event that more than 50% of the entire questionnaire outcome data are missing, an approach will be discussed and agreed on with the DMC, including, depending on the pattern of missing data, reducing the study to a parallel-arm analysis using first-period data alone.

## Ethics and dissemination

Full research ethics committee (REC), Health Research Authority and MHRA approval has been granted (REC reference: 20/SC/0416, approved by South Central-Oxford B REC on 6 January 2021). The current version of the protocol is V.3.1, dated 14 November 2022.

Participants provide written informed consent to take part. Their right to refuse participation or request withdrawal is respected at any stage. Following participation, routine clinical care will continue for each participant, without an open-label extension.

The results of the trial will be disseminated by publication regardless of the direction or magnitude of effect. The detailed statistical analysis plan will also be published.

### Current study status

This draft protocol has been aided by the 2013 Standard Protocol Items: Recommendations for Interventional Trials guidelines.^53^

The first participant was randomised on 30 June 2021. Recruitment is expected to take 50 months in total, finishing in autumn 2025.

The course of the trial was interrupted by the COVID-19 pandemic restrictions, and then a temporary pause in trial recruitment related to recurrent IMP supply issues. The study team remained blinded during this period. There were no safety concerns. Later, national supply chain difficulties with commercially available atomoxetine led to intermittent disruptions to recruitment, but no disruption to individual participants’ supply.

As of 16 May 2025, 76 participants have been screened and entered the treatment phase (visit 2) of the study. During the pause for IMP supply issues, 7 participants who initially consented and were randomised in the trial declined or were unable to resume participation in 2023 due to their disease progression or death.

## Supplementary material

10.1136/bmjopen-2025-099577 online supplemental material 1
