# Supplementary material for: Noradrenaline for progressive supranuclear palsy syndromes (NORAPS): a randomised, double-blind, placebo-controlled, crossover Phase IIb clinical trial evaluating the efficacy and safety of oral atomoxetine for treating cognitive and behavioural changes in people with progressive supranuclear palsy syndromes in the UK
Source: BMJ Open. 2025 Jul 28;15(7):e099577. doi: 10.1136/bmjopen-2025-099577 (PMC12306469; doi:10.1136/bmjopen-2025-099577)
Supplement: online supplemental material 1 [file bmjopen-15-7-s001.pdf]

Department of Clinical Neurosciences

Information Sheet and Informed Consent Form for Research Partners

Noradrenaline for the Treatment of Cognitive and Behavioural Change in Progressive Supranuclear Palsy Syndromes (NORAPS)

- You are invited to take part in a research trial.
- Before deciding whether to take part, you need to understand why this research is being done and what it involves.
- Please ask if anything is not clear or if you would like more information.
- Take time to read the following information carefully and talk to others about the trial if you wish.
- Take time to decide whether or not you wish to take part.

Section 1: Purpose of the trial and what will happen

What is the purpose of the trial?

Progressive Supranuclear Palsy (PSP) changes cognition (thinking) and behaviour, as well as movement and balance. This is because of changes in brain function. Healthy brain function depends on naturally occurring chemicals ('neurotransmitters') that enable brain cells to communicate with each other. We believe that the loss of the chemical called Noradrenaline causes some of the cognitive and behavioural changes in PSP. This trial looks at the effects of a medication called Atomoxetine, which boosts Noradrenaline in the brain.

What is the medication being tested?

Atomoxetine is a medication that increases the levels of Noradrenaline in the brain. It is licensed in the UK and the USA for treatment of Attention Deficit Hyperactivity Disorder (ADHD), and has been used in many research studies in healthy adults here in the UK and elsewhere. This medication has also been shown to improve cognitive and

behavioural changes in some patients with Parkinson's disease. In this trial, Atomoxetine will be given as an oral solution, using the standard starting oral dose for adults (40mg) for 8 weeks.

Why have I been invited?

You have been invited to take part because you have regular contact with a patient who has PSP, and they have suggested that you might be able to provide information about what you notice about the way their behaviour changes. Because self-report can be influenced, for example by the motivation of presenting oneself positively, it is important for information about behavioural changes to be collected from the patient but also someone who is in regular contact with them. The information you will give will therefore be used in addition to the information the patient will give about themselves.
We believe Atomoxetine may be a suitable treatment to help people with PSP. We plan to enrol 84 patients across the UK with PSP who have a “research partner” (a family member, close friend or carer).

Do I have to take part?

No. Participating in this trial is voluntary. If you decide to participate you will be asked to sign a Consent Form, however you are still free to change your mind and leave the trial at any time without giving a reason.

What will happen to me if I take part?

You will be asked to accompany the patient to the clinic for **6 visits** over 6 months.
Depending on your local hospital’s policy about COVID-19, you might be asked to go to the hospital before the trial visits to do a COVID-19 swab test in order to confirm that you don’t have the virus.
Sessions may last up to five hours, including periods of rest.

	**Visit 1 - Baseline (up to 28 days before the patient starts trial treatment)**<br>• We will ask you and the patient to sign consent forms to take part in the trial.<br>• A doctor will assess the patient (not you). They may need a heart tracing test (ECG) and have a blood test.<br><br>After the visit the trial doctor or a member of the trial team will let the patient know if they are eligible and can safely continue in the trial.

**Week 0**	**Visit 2 - The start of the first treatment phase**<br>• You will be asked to attend clinic again within 28 days from the baseline visit.<br>• You will be asked to complete some questionnaires about the patient.<br>• A doctor will assess the patient, and they will complete some questionnaires and memory tests, before taking their first dose of medication. We will monitor them for 3 hours, with a blood sample and ECG.<br>• After visit 2, the patient will take the trial medication once in the morning for 8 weeks. We will ask you (or the patient's carer if they have one and is not yourself) to help them do this and record it in a diary.<br>The medication might be slightly cloudy in appearance or have some sediment, this is nothing to worry about and does not need to be reported to the research team
**Week 4**	2-4 weeks **after starting the trial medication** we will telephone the patient or you/carer to check for any side effects, to confirm that they are taking the trial medication correctly, and to arrange for more medication to be collected by the patient or you/carer at the hospital pharmacy. They may be asked to return the first bottle of trial medication at this time to the pharmacy department.
**Week 8**	**Visit 3 - The end of the first treatment phase**<br>• You and the patient will be asked to attend clinic again after 8 weeks.<br>• A doctor will perform a medical assessment of the patient.<br>• You will be asked to complete some questionnaires about the patient.<br>• The patient will be asked to return all the bottles of trial medication (empty or not) to the pharmacy department, and their diary to the trial team at this visit.<br>• After visit 3, the patient will have a two weeks treatment break.
**Week 10**	**Visit 4 - The start of the second treatment phase**<br>• You and the patient will be asked to attend clinic again after two weeks.<br>• A doctor will perform a medical assessment of the patient.<br>• You will be asked to complete some questionnaires about the patient.<br>• The patient will be given the trial medication and take the first dose in clinic.<br>• We will monitor them for 3 hours, with a blood sample and ECG.<br>• The patient will then take the trial medication once in the morning for 8 weeks. We will ask you/the carer to help them do this and record it in a book.
**Week 14**	4 weeks after starting **the second treatment phase** we will telephone to check for any side effects, confirm that they are taking it correctly, and arrange for more medication to be collected by the patient or you/carer at the hospital pharmacy. The patient or you may be asked to return the first bottle of trial medication to the pharmacy at this time.
**Week 18**	**Visit 5 - The end of the second treatment phase**<br>• You and the patient will be asked to attend clinic again after 8 weeks.<br>• A doctor will perform a medical assessment of the patient.

	• You will be asked to complete some questionnaires about the patient.<br>• The patient or you will be asked to return all the bottles of trial medication (empty or not) to the pharmacy department, and their diary to the trial team at this visit.
**Week 22**	**Visit 6 - End of Trial Assessment**<br>• You and the patient will be asked to attend clinic again four weeks after they finish the trial medication.<br>• A doctor will perform a medical assessment of the patient.<br>• You will be asked to complete some questionnaires about the patient.

Note that you will be expected to attend the visits with the patient but if for exceptional circumstances (e.g. health reasons) you cannot attend a visit (even the first one), it will be possible for the research team to send you the trial documents to be completed.

What are the possible benefits of taking part?

Information collected as part of your participation in this trial may benefit patients with PSP in the future. You will also have the pleasure of knowing that you have made contribution to our understanding of PSP and the effects of the trial medication on the brain.

You will not receive any payment for participating in this trial, however we will reimburse expenses required for any inconvenience associated with the trial.

This includes travel expenses (40p /mile or the use of public transport with receipt) for you and the patient. If needed, transport can also be arranged (Taxi). Refreshments will also be provided during all the visits. When needed, a light meal will also be provided or reimbursed (with receipts - £10 to £15 per visit).

What are the possible disadvantages and risks of taking part?

You will be asked to accompany the patient to the clinic for 5 visits over 5 months during their treatment. During the questionnaires, we will only ask you questions which we would like you to answer as best as you can. However, we appreciate for some people this may be upsetting.

If you are unhappy about any part of the trial, you are encouraged to discuss this with the local trial team or with the Patient Assistance and Liaison Services (PALS) at your local Hospital. Normal legal processes are also open to you. Insurance has been arranged in the unlikely event of any loss or injury.

Who has reviewed this trial?

This trial has been approved by the South Central - Oxford B Research Ethics Committee. The Medicines and Healthcare Products Regulatory Agency (MHRA) who are responsible for regulating medicines in the UK have also approved this trial.

What if I decide I no longer wish to participate in the trial?

You are free to stop taking part in the trial at any time. If you choose to take part but then change your mind, any information you have agreed to provide until that point will be kept but no further information collected. Not continuing in this trial will not affect the patient’s care in any way.

Will my taking part in this trial be kept confidential?

Yes.
Cambridge University Hospitals NHS Foundation Trust (CUH) and The University of Cambridge are the joint Sponsors for this clinical trial based in the United Kingdom. They will be using information from you in order to undertake this trial and will act as the data controller for this trial. This means that they are responsible for looking after your information and using it properly. The Sponsor organisations will keep identifiable information about you for 5 years after the trial has finished to allow the trial to be reviewed by the authorities after it is finished.

Your rights to access, change or move your information are limited, as the Sponsor organisations need to manage your information in specific ways in order for the research to be reliable and accurate. To safeguard your rights, we will use the minimum personally-identifiable information possible.

You can find out more about how the Sponsors use your information using the information below:

- For Cambridge University Hospitals NHS Foundation Trust, please visit: https://www.cuh.nhs.uk/corporate-information/about-us/our-responsibilities/looking-after-your-information, or email the Data Protection Officer at: cuh.gdpr@nhs.net

- For University of Cambridge, please visit:
https://www.medschl.cam.ac.uk/research/information-governance/, or email the Information Governance team at: researchgovernance@medschl.cam.ac.uk

The University of Cambridge will keep your name and contact details to contact you about this trial, and make sure that relevant information about the trial is recorded, and to oversee the quality of the trial. Certain individuals from the Sponsor(s), coordinating centre and regulatory organisations may look at your research records to check the accuracy of this trial. The Sponsors will only receive information without any identifying information.
The University of Cambridge will keep identifiable information about you from this trial for 5 years after the trial has finished.

All information collected about you as a result of your participation in the trial will be kept strictly confidential. Your personal information will be kept in a secured file and be treated in the strictest confidence.

Once you have agreed to participate in this trial you will be allocated a Trial ID Number. This is a unique trial number which will be used on all your trial documentation.

The people who analyse the information will not be able to identify you and will not be able to find out your name, or contact details. Only anonymous trial data, without any personal information will be published at the end of the trial.

A copy of the consent form with your name and signature will also be sent to Norwich Clinical Trials Unit (coordinating centre). The consent form will be checked to ensure that it has been correctly completed and then destroyed.

When you agree to take part in this trial, the information you gave about yourself and the patient may be provided to researchers running other research studies in this organisation and in other organisations. These organisations may be universities, NHS organisations or companies involved in health and care research in this country or

abroad. Your information/ the information you gave about the patient will only be used by organisations and researchers to conduct research in accordance with the UK Policy Framework for Health and Social Care Research. This information will not identify you or the patient and will not be combined with other information in a way that could identify you or the patient. The information will only be used for the purpose of health and care research, and cannot be used to contact you.

What will happen to the results of the trial?

When the results of this trial are available they may be published in peer reviewed medical journals and used for medical presentations and conferences but the results will only contain anonymous information and trial participants will not be identifiable. If you would like to obtain a copy of the published results please contact your trial doctor directly who will be able to arrange this for you.

Who is organising and funding the trial?

This trial is sponsored by Cambridge University Hospitals NHS Foundation Trust and the University of Cambridge. The trial is being funded by the Medical Research Council, Cambridge Centre for Parkinson's Plus. The trial will be coordinated by the Norwich Clinical Trials Unit.

Further information and contact details

Contacts for more information:

Professor James Rowe (Chief Investigator)
01223 760695 james.rowe@mrc-cbu.cam.ac.uk

Dr Robert Durcan (Sub Investigator)
01223760699 | rd693@medschl.cam.ac.uk

Hugo Paula (Research Nurse)
01223764047 | hp487@medschl.cam.ac.uk

Out of Hours, please contact the local investigators on 01223 245151 (Addenbrooke's contact centre). Please explain that this is an emergency related to the NORAPS trial so that the operator knows who to contact.

In the unlikely event of anything untoward happening, you may complain either directly to Professor Rowe (Chief Investigator), or through the Patient Liaison and Advisory Service (PALS) at Addenbrooke's Hospital. Box 53, Cambridge University Hospitals, Cambridge Biomedical Campus, Hills Road, Cambridge, CB2 0QQ. 01223 216756. cuh.pals@nhs.net.

RESEARCH PARTNER INFORMED CONSENT FORM

Noreadrenaline for the Treatment of Cognitive and Behavioural Change in Progressive Supranuclear Palsy Syndromes (NORAPS).

Research partner Identification Number ☐ — ☐☐☐ — ☐

Site ID Patient ID SI ID

Please initial each box

Principal Investigator: ____________________

1. I confirm that I have read and understand the information sheet for Research Partner dated __ 25 Nov 2022__ (version __2.2_) for the above trial and I confirm that the trial procedures and information have been explained to me. I have had the opportunity to ask questions and I am satisfied with the answers and explanations provided.	
2. I understand that my participation is voluntary and that I am free to withdraw at any time, without giving any reason, without my legal rights, or the medical care of the patient I accompany/care for being affected.	
3. I understand that personal information about me will be collected and used in accordance with this information sheet. This information will be kept in the strictest confidence and none of my personal data will be published.	
4. I give permission for a copy of this consent form to be kept confidentially and securely by the coordinating centre until it has been reviewed.	
5. I agree to my personal contact details being documented for the purpose of sending trial documents by email or post	

6. I agree to complete all trial questionnaires to the best of my ability.	
7. I agree for my data to be stored securely in a coded form.	
8. I understand that relevant section of data collected during the trial, may be looked at by responsible individuals from the research team, regulatory authorities, sponsor or from the NHS trust where it is relevant to my taking part in this research. I give permission for these individuals to have access to my records.	
9. I agree to take part in the above trial.	

Name of Research partner   Date (dd/mmm/yyyy)   Signature

Name of person taking consent   Date (dd/mmm/yyyy)   Signature

Original: Investigator site file. Copies: 1 to NCTU Trial Office, 1 to Research partner, 1 to Patient Medical Notes
