# Supplementary material for: Noradrenaline for progressive supranuclear palsy syndromes (NORAPS): a randomised, double-blind, placebo-controlled, crossover Phase IIb clinical trial evaluating the efficacy and safety of oral atomoxetine for treating cognitive and behavioural changes in people with progressive supranuclear palsy syndromes in the UK
Source: BMJ Open. 2025 Jul 28;15(7):e099577. doi: 10.1136/bmjopen-2025-099577 (PMC12306469; doi:10.1136/bmjopen-2025-099577)
Supplement: Supplementary file 2 [file bmjopen-2025-099577.reviewer_comments.pdf]

PEER REVIEW HISTORY

BMJ Open publishes all reviews undertaken for accepted manuscripts. Reviewers are asked to complete a checklist review form and are provided with free text boxes to elaborate on their assessment. These free text comments are reproduced below.

ARTICLE DETAILS

Title (Provisional)

Noradrenaline for Progressive Supranuclear Palsy Syndromes (NORAPS): a Randomised, Double-Blind, Placebo-Controlled, Crossover Design, Phase IIb Clinical Trial to Evaluate the Efficacy and Safety of Oral Atomoxetine for the Treatment of Cognitive and Behavioural Change in People with Progressive Supranuclear Palsy Syndromes in the UK.

Authors

Durcan, Robert; Paula, Hugo; Ghosh, Boyd C P; Street, Duncan; High, Juliet; McAlister, Colin; Shepstone, Lee; Russell, Charlotte; Grant, Kelly; Igosheva, Natalia; Rodgers, Christopher T.; Jones, Simon P; Ye, Rong; Kobylecki, Christopher; Church, Alistair; Antoniades, Chrystalina; Marshall, Vicky; Passamonti, Luca; Rowe, James B

VERSION 1 - REVIEW

**Reviewer**	**1**
**Name**	**Vivash, Lucy**
**Affiliation**	**Monash University, Neurosciences**
**Date**	**26-Mar-2025**
**COI**	**None**

This is a randomised, double blind cross over study of atomoxetine for the treatment of cogntiive and behavioural symptoms in patients with PSP.

It is both a safety and tolerability and efficacy study - using subscores of the revised cambridge behavioural inventory for the efficacy measures.

Overall the paper is well presented - I have a couple of suggestions on moving sections around to improve flow, and adding a few additional details (see below). The only major concern I have is that there are no details on the statistics (and power) for the predefined outcomes. Whilst I understand that a SAP is being developed, I consider it important that the statistical analyses of the outcomes (as indicated in the study protocol) are detailed in the protocol paper.

Minor points:

- Page 7, lines 33-37: Could the authors provide more details on the fMRI and ASL acquisitions. Also, NODDI is an analysis method for DWI, please provide details of the acquisition sequence.

- Page 7, line 5. How much blood is being taken? Also, is there only one PK sample at 2 hours post dose?

- Page 10, line 25: Consider moving the inclusion/exclusion criteria to before the details of each visit so that it is clear who meets eligibility at the screening visit.

- Page 11, line 55: How does randomisation occur?

- Page 13/page 14, line 42: How will the outcome measures be analysed and reported?

- Page 15, line 27: Can an update on the current enrolment, completion and early discontinuation numbers be provided?

**Reviewer**	**2**
**Name**	**Kaushal, Himanshu**
**Affiliation**	**Mahatma Gandhi Medical College and Hospital, Neurology**
**Date**	**27-Mar-2025**
**COI**	**None**

---

1. Please provide limitations and strengths of the proposed study

2. Kindly attached Plagiarism report

3. • To assess the efficacy of atomoxetine compared to placebo

on behaviour and cognition.

To estimate the efficacy of atomoxetine compared to

placebo on cognitive and behavioural functioning.

VERSION 1 - AUTHOR RESPONSE

Comments to Reviewer 1:

(Dr. Lucy Vivash, Monash University)

This is a randomised, double blind cross over study of atomoxetine for the treatment of cogntiive and behavioural symptoms in patients with PSP.

It is both a safety and tolerability and efficacy study - using subscores of the revised cambridge behavioural inventory for the efficacy measures.

Overall the paper is well presented - I have a couple of suggestions on moving sections

around to improve flow, and adding a few additional details (see below). The only major concern I have is that there are no details on the statistics (and power) for the predefined outcomes. Whilst I understand that a SAP is being developed, I consider it important that the statistical analyses of the outcomes (as indicated in the study protocol) are detailed in the protocol paper.

Response: Thank you for the positive feedback. We agree with the reviewer and have amended the text accordingly in the revised version of the manuscript on page 13 and 14 to include a summary statistical plan of analysis. Our power calculation is found in sample size section on page 12.

Minor points:

- Page 7, lines 33-37: Could the authors provide more details on the fMRI and ASL acquisitions. Also, NODDI is an analysis method for DWI, please provide details of the acquisition sequence.

Response: Thank you. We confirm that the NODDI sequence is a version of diffusion weighted imaging and include details of this sequence, and the ASL, on page 7.

- Page 7, line 5. How much blood is being taken? Also, is there only one PK sample at 2 hours post dose?

Response: Thank you. This has been clarified on page 8. We take blood after dosing at the beginning of each treatment phase and end of each treatment phase (four blood draws), in addition to safety bloods at the screening visit and close-out visit). This is partly for confirmation of successful randomisation, and partly to estimate the variation in plasma levels between participants (PK), following the standard dose at Visit 2 and 4. Previous day curve studies with hourly sampling in older adults with parkinsonism have confirmed that 2 hours is suitable to estimate peak concentration.

- Page 10, line 25: Consider moving the inclusion/exclusion criteria to before the details of each visit so that it is clear who meets eligibility at the screening visit.

Response: Thank you for this helpful suggestion. We agree and have amended the text accordingly.

- Page 11, line 55: How does randomisation occur?

Response: Thank you. Randomisation uses a web-based process, managed centrally by the Norwich clinical trials unit for all sites. This has been amended in the text on page 10.

- Page 13/page 14, line 42: How will the outcome measures be analysed and reported?

Response: Thank you. Clarification of both outcome measures has been included on page 13. The proposed reporting of the trial is outlined in the Ethics and Dissemination section on page 14.

- Page 15, line 27: Can an update on the current enrolment, completion and early discontinuation numbers be provided?

Response: Thank you. We have included this information on page 15 of the updated manuscript.

Comments to Reviewer 2
Dr. Himanshu Kaushal, Mahatma Gandhi Medical College and Hospital, Mahatma Gandhi University of Medical Sciences & Technology

1. Please provide limitations and strengths of the proposed study

Response: Thank you. This has been provided on page 2 of the revised manuscript.

2. Kindly attached Plagiarism report

Response: Thank you for outlining this omission. It was not intentional.

3. • To assess the efficacy of atomoxetine compared to placebo
on behaviour and cognition.
To estimate the efficacy of atomoxetine compared to
placebo on cognitive and behavioural functioning.

Response: Thank you for the comments. We would in general terms be happy to amend the wording to include the phrase "on cognitive and behavioural functioning **". However, given that the trial has started, we are reluctant to change the wording of the primary endpoint, and minimise deviations from the trial registry wording. On the suggestion of "estimate" versus "assess", please note that the statement of primary objective in the manuscript (page 3, and p14) already uses the word** "estimate". **Elsewhere, including the secondary objectives, we use "assess" in line with standard usage regarding cognitive tasks and behavioural observations, where these are taken directly from the participant or their informant. The word "estimate" would be most appropriate if indirect or imputation-based methods were used, but where a direct measurement is planned, it is at least as appropriate to refer to assessment as estimation.**
